# Combination of Texture-Induced Oral Processing and Vegetable Preload Strategy Reduced Glycemic Excursion but Decreased Insulin Sensitivity

**DOI:** 10.3390/nu14071318

**Published:** 2022-03-22

**Authors:** Yixue Wu, Zhihong Fan, Xinling Lou, Wenqi Zhao, Xuejiao Lu, Jiahui Hu, Yue Han, Anshu Liu

**Affiliations:** 1 College of Food Science and Nutritional Engineering, China Agricultural University, Beijing 100083, China; xiaoc0105@126.com (Y.W.); xinlinglou@cau.edu.cn (X.L.); zhaowenqi@cau.edu.cn (W.Z.); feirlu@163.com (X.L.); hujiahui1023@126.com (J.H.); hanyuecau@163.com (Y.H.); liuanshu@cau.edu.cn (A.L.); 2 Key Laboratory of Precision Nutrition and Food Quality, Department of Nutrition and Health, China Agricultural University, Beijing 100083, China

**Keywords:** oral processing, broccoli, glycemic response, insulinemic response, food intake sequence

## Abstract

This study aimed to investigate the effect of the oral processing of vegetables induced by texture modification on acute postprandial glycemic response (GR) and insulin response (IR) when co-ingested and ingested prior to a rice meal. In a randomized crossover trial, 14 healthy female subjects consumed (1) co-ingestion of soft broccoli and rice (SR); (2) co-ingestion of hard broccoli and rice (HR); (3) soft broccoli prior to rice (S+R); (4) hard broccoli prior to rice (H+R); (5) rice (R). Postprandial GR and IR was compared between test meals over a period of 180-min, and the oral processing behaviors were measured for each test food samples. Hard broccoli was observed to have a higher mastication time and chews than soft broccoli. All the broccoli meals resulted in reduced incremental peak glucose (IPG) and an increased incremental area under the insulin curve in 180 min (iAUC_0–180_) compared with R. The S+R curbed the IPG by 40% with comparable HOMA-IR AUC_0–180_ compared with R, while the H+R elevated the HOMA-IR AUC_0–180_ by 62% more than that of R. In conclusion, the soft broccoli intake prior to a rice meal effectively attenuated postprandial GR, without lowering insulin sensitivity as its hard counterpart did.

## 1. Introduction

Postprandial glycemic response (GR) is an important health concern well acknowledged by the public, as sustained hyperglycemia is a risk factor of type 2 diabetes (T2D) [1]. Sufficient vegetable intake has been associated with reduced risk of T2D [2], especially the intake of green leafy vegetables [3] and cruciferous vegetables [4,5]. Addition of vegetables to high glycemic index (GI) foods such as refined white rice [6] has been proved to attenuate postprandial (GR) and insulin response (IR) indicating an elevated insulin sensitivity [7,8].

The effect of vegetable intake on glucose homeostasis and insulin sensitivity are reported to be attributed to the dietary fiber [9,10], phytochemicals [11] and the minerals counteracting the excess acid load [12,13]. However, the natural structure of the vegetable itself might also be a key factor in curbing postprandial GR. A previous study has shown that non-homogenized cooked vegetables were more effective in mitigating the postprandial GR and glycemic index (GI) compared with homogenized counterparts with comparable contents of polyphenols and dietary fiber [14]. This research suggested a potential association between the oral processing potential and the glycemic mitigation effect of vegetables, mediated by its texture properties.

Mastication has a major role in reducing the particle size of a food to form a bolus for swallowing. The extent of mastication decides the bolus particle size and the accessibility of enzymes to food substrates [15]. Previous research demonstrated that oral processing might contribute to variations in postprandial GR [16,17,18], insulin [19] and satiation-related entero-endocrine secretion [20]. Thoroughly chewed carbohydrate foods have been observed to have a higher postprandial GR compared to those swallowed whole or normally chewed [21]. A number of relevant studies focused on the impact of mastication variation on GR and IR of carbohydrate-based food [22,23,24], but it remains unclear whether the effect of the oral processing variation of non-carbohydrate foods, such as vegetables, can influence postprandial GR and IR in a mixed meal.

Oral processing behaviors are elicited by a food’s physical and mechanical properties [25,26,27,28], and the individuals adjust their mastication in response to the food textures in a natural way [29]. A number of studies investigated the influence of texture on postprandial GR [30,31], but few studies have reported the impact of oral processing behaviors on postprandial GR and IR when consuming texture-modified vegetable samples in a meal. In addition, taking vegetables prior to the ingestion of high glycemic carbohydrate foods such as rice has been proved to be able to attenuate the postprandial GR [32,33,34]. However, the acute outcome of a combination of modified oral processing and meal intake sequence has yet to be explored in terms of the postprandial GR and IR.

Polished rice is a world-wide popular staple food and a major contributor to daily glycemic load in many regions. The japonica type of rice, prevalent in northeast Asia, is regarded as a typical high glycemic food [35]. In the Asian food culture, rice is usually consumed with dishes including broccoli [36]. Broccoli is one of the most accepted nutrient-dense vegetables in the world. As a side dish or a vegetable dish, broccoli had the advantage of being able to be prepared into textures of various degree of hardness.

In this study, cooked broccoli of soft and hard texture was ingested with or prior to a rice meal to evaluate the impact of oral processing and meal sequence on GR and IR. The research hypotheses were:

**Hypothesis** **1 (H1).** *The hard broccoli would elicit more chews and lower the postprandial glycemic excursion more effectively compared with the soft broccoli.*

**Hypothesis** **2 (H2).** *The combination of chewy texture and early ingestion of broccoli would curb the postprandial glycemic excursion better than its co-ingestion counterpart.*

**Hypothesis** **3 (H3).** *The increased oro-sensory exposure (OSE) induced by hard broccoli would not elevate the insulin peak value.*

## 2. Materials and Methods

### 2.1. Subjects

Healthy female adult volunteers aged 18–30 years were recruited through online advertisements on social media channels and bulletin boards. Participants underwent an initial screening online on the basis of the inclusion criteria: (1) body mass index (BMI) of 18.5 to 23.9 kg/m^2^; (2) with healthy dentition and the ability to bite, chew and swallow normally; (3) no reported food allergies or intolerance to the test meals; (4) weight stable (±2 kg) within the last six months; (5) no metabolic disease or ongoing medical treatment; (6) no diagnosed digestive system diseases, or self-reported frequent gastrointestinal upset; (7) regular sleep and meal consumption; (8) a regular menstrual cycle without pregnancy and lactation; (9) no dependency on alcohol or tobacco.

The power calculation was performed by PASS 15 Power Analysis and Sample Size software (NCSS, Kaysville, UT, USA). The study recruited a minimum sample size of 12 to observe a difference between iAUC glucose and oral processing behaviors with 80% power and 5% significance level [37]. With a 30% anticipated attrition rate, the number of recruited participants was extended to 15.

A further health screen was carried out in the laboratory to assess eligibility, including a duplicated oral glucose tolerance test (OGTT), measurements of weight, height, BMI, body fat, waist circumference and blood pressure. The study was conducted in accordance with the Declaration of Helsinki and was approved by the Ethics Committee of the China Agricultural University (ethics number CAUHR-2021010).

### 2.2. Study Design

In this crossover, randomized and acute feeding trial, subjects attended a mastication session and five test-meal sessions separated by at least three days as adequate washout at the China Agricultural University. Each participant attended the mastication session of 20 min during which oral processing behaviors were determined using video recordings. The test-meal sessions assigned subjects to five test meals in a randomized order generated by the computer. No test session was carried out in the three days before the menstrual period nor during the first three days of the period to prevent the possible impact of premenstrual syndrome.

On the day prior to each test session, subjects were instructed to have the identical dinner prior to each test day at school canteens and maintain their usual physical habits while not consuming any broccoli, and avoiding coffee, tea and alcohol consumption, strenuous exercise, and a later bedtime.

**Figure 1:**
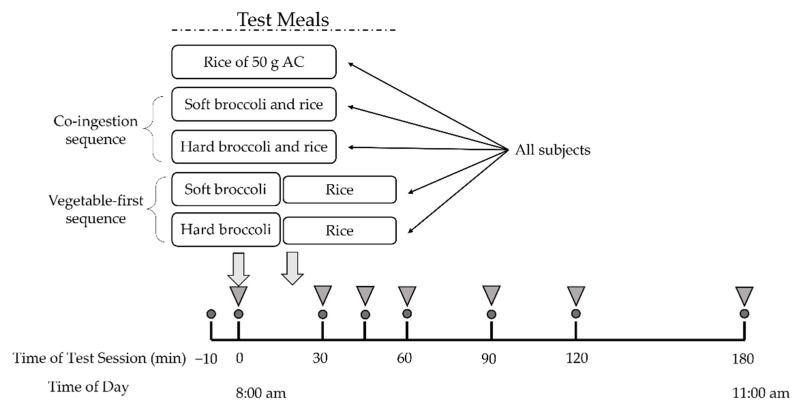
Study experimental design. All subjects underwent five test meal studies. Circles indicate times of blood glucose measurement. Inverted triangles indicate blood collection for insulin measurement. AC, available carbohydrate.

Participants fasted for 12 h overnight and arrived at the laboratory at 7:50 a.m. The test began at 8:00 a.m. and the moment of first bite of test meal was defined as 0 min. After a short rest, two fasting plasma glucose samples was collected ten minutes before the test (−10 min) and at 0 min. Subjects were subsequently asked to consume the provided broccoli-containing test meals within 30 min or the rice meal within 10 min. Then they were supplied with 150 mL of plain water at room temperature at 120 min and were required to finish it before the end of the test session. Postprandial blood samples were obtained by finger prick for 180 min with a continuous sedentary condition of the participants (Figure 1). 

### 2.3. Meals

Subjects were served two test meals to be consumed in different sequences as well as a rice reference meal. The five test meals and their food intake sequence were as followed: (1) co-ingestion of soft broccoli and rice (SR); (2) co-ingestion of hard broccoli and rice (HR); (3) soft broccoli ingested prior to rice (S+R); (4) hard broccoli ingested prior to rice (H+R); (5) pure rice as reference meal (R). Broccoli, served at 0 min, was consumed within 20 min and rice was subsequently served at the time when broccoli consumption finished and was consumed within 10 min in the S+R and H+R group. The broccoli and rice were freshly prepared on the morning of the trial days and served to the subjects within 30 min of preparation.

Broccoli (*Brassica oleracea var.* italica) was bought from the local supermarket. Fresh broccoli heads were cut into florets with approximately 2 cm stem length and 3 cm florets diameter. The florets were mixed thoroughly with 62.5 g plain water in Snap Boxes, 250 g raw broccoli florets were cooked in microwave for 2.5 min (hard broccoli, HB) and 4.5 min (soft broccoli, SB). The cooking times of SB and HB were screened through a pilot study of sensory rating for broccoli texture where SB and HB differed in hardness with comparable pleasantness and acceptance. The nutritional composition of the test meals is shown in Table 1.

### 2.4. Texture Analysis

Instrumental texture parameters of broccoli were measured by puncture and by shear using TA.XT Plus Texture Analyzer (Lotun Science Co., Ltd., Beijing, China) equipped with 2-mm-diameater probe and HDP/BS probe [38]. Measurements were made on stem samples of broccoli for a puncture test (10 mm diameter × 50 mm) and shear test (10 mm diameter × 200 mm). Each test was performed with a minimum of eight replicates on a batch of cooked broccoli at room temperature. For the puncture test, the probe penetrated the stem slice at a constant speed of 1 mm/s to a depth of 10 mm and the puncture force and flexibility (distance of trigger point to break point) were recorded. For the shear test, the probe sheared the cylindrical stem at a constant speed of 1 mm/s to a depth of 15 mm and the shear force, toughness (incremental areas under the force-distance curve) and brittleness (number of incremental peaks) were obtained.

### 2.5. Analysis of Oral Processing Behaviors

Oral processing behaviors were characterized in duplicate by webcam (Philips SPL6506BM) video recordings described previously by Arianne et al. [39]. The samples were prepared in the same way in the test meal sessions. Rice samples were served at 5.0 g and broccoli samples were served at approximately 10 g in cooked weight. Recordings of each sample consumption were coded using a Kinovea software 0.8.15 with a standardized behavioral coding approach based on Arianne et al. [39]. Characteristics of eating patterns such as eating rate (the mass of food consumed per minute, g/min); chews per bite; chews per gram were calculated by the recorded frequencies of key ‘point’ events (bites, chews and swallows) and duration of a single ‘continuous’ event (total mastication time).

### 2.6. Blood Collection and Metabolite Measurements

Blood glucose concentrations (the second drop of blood) were measured by the glucose oxidase method on the baseline (−10 and 0 min) and at 15; 30; 45; 60; 90; 120; 150; and 180 min after the start of the meal with a glucometer (LifeScan Inc., Milpitas, CA, USA).

At each time point, 150 μL of capillary blood (obtained from finger pricks) was collected into EDTA K2-coated centrifuge tubes (WanDGL Ltd., Jinan, China) and stored in crushed ice immediately. The tubes with blood were centrifuged at 1000× *g* for 15 min with 60 μL supernatant plasma pipetted into Eppendorf tubes and stored at −80 °C until being analyzed later. Plasma insulin concentrations were determined using an ELISA-based test kit (JunLB Ltd., Beijing, China).

### 2.7. Statistical Analysis

The oral processing data were based on the recorded frequencies of bites, chews and swallows and duration of food in mouth. Eating rate (g/min), chews per bite, chews per gram, chews’ rate (chews/s) and total mastication time(s) were calculated.

The fasting glucose concentration was taken as the average of the glucose level at −10 min and 0 min. Indices of glycemic variability included the incremental peak glucose concentrations (IPG), time to IPG, and the positive increments under the curve of postprandial GRs (iAUC) calculated by trapezoid summation [40]. The insulin data were based on the percent change of insulin relative to the fasting insulin value to eliminate inter-personal variability. The positive incremental areas under the curve (iAUC) of postprandial insulin responses, the incremental peak of insulin (IPI) and the time of IPI were calculated. Homeostatic model (HOMA-IR) test was applied to assess insulin resistance [41] during both the fasting and the postprandial periods. Postprandial homeostatic model areas under the curve (HOMA-IR AUC) were calculated by the product of insulin and glucose AUCs divided by 22.5 [42].

With the preliminary collation of original data in Microsoft Excel spreadsheets, data of glucose iAUCi; insulin iAUCi; IPG; and IPI greater than 2 SDs were considered outliers and excluded [43]. The effects of treatment × time on GR and insulin response were tested with two-factor repeated-measures ANOVA. Effects of test meals’ characteristic values of texture, mastication behaviors, GR and insulin response mentioned above were assessed by one-way analysis of variance ANOVA and Duncan’s multiple range test. All results were reported as mean values and standard deviation (SD) or standard error of means (SEM) as appropriate with *p* < 0.05 considered as statistically significant. The statistical analysis was performed by the SPSS version 23.0 (SPSS Inc., Chicago, IL, USA) and the figures were generated from Origin 2022 (OriginLab Inc., Northampton, MA, USA).

## 3. Results

### 3.1. Subject Characteristics

**Figure 2:**
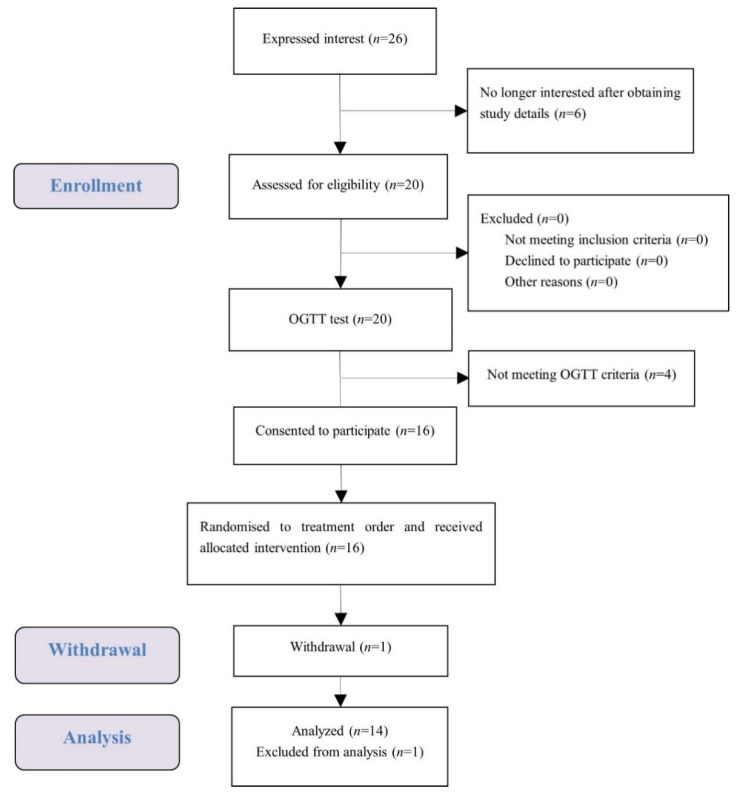
Consolidated standards of reporting trial (CONSORT) flow diagram of the study subjects.

A total of 16 female university students met the health and OGTT criteria, and one of them dropped out (Figure 2). The test data of 14 subjects were put into analysis with data of one subject considered as an outlier and excluded. Demographic characteristics of the subjects are presented in Table 2. Average measures were within the normal range for anthropometry, blood pressure, glucose, and insulin. No subject reported any adverse effect or unpleasant feeling during all the test sessions.

### 3.2. Instrumental Texture Characteristics of Broccoli

The instrumental texture characteristics of broccoli are shown in Table 3. Both puncture force and the shear force of HB were significantly higher than SB, which confirmed the result of the pilot screening. Significantly higher brittleness was detected in HB than SB.

### 3.3. Oral Processing Behaviours

The oral processing characteristics for each test sample are summarized in Table 4. Average chew rate (chews/s) did not differ significantly between rice, SB and HB. Hard broccoli was consumed for 26% longer seconds than soft broccoli, and 58% longer seconds than rice (*p* < 0.05). The hard broccoli was also eaten with 17 additional chews compared to the soft broccoli and 26 additional chews compared to the rice reference (*p* < 0.05). Soft broccoli mastication had 70% less chews per gram than rice with the highest eating rate (*p* < 0.05).

### 3.4. Postprandial Glucose and Insulin Response

**Figure 3:**
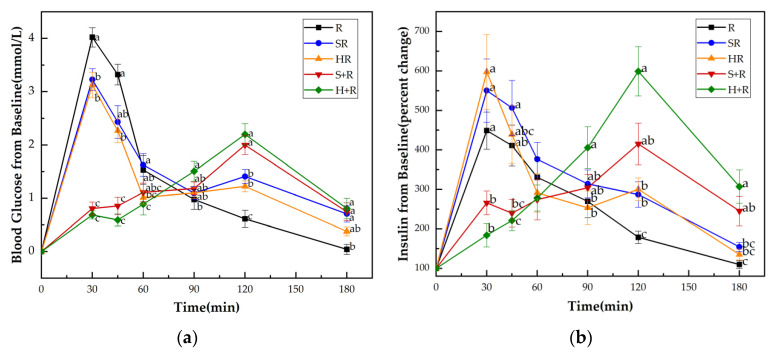
(**a**) Blood glucose concentration changes from baseline for test meals; (**b**) Insulin concentration changes from baseline for test meals. R, rice; SR, co-ingestion of soft broccoli and rice; HR, co-ingestion of hard broccoli and rice; S+R, soft broccoli ingested prior to rice; H+R, hard broccoli ingested prior to rice. Values are the mean changes in blood glucose levels (**a**) and percent changes in insulin levels (**b**) from baseline, *n* = 14, with their standard errors represented by vertical bars. Different letters indicate significant differences between means (*p* < 0.05).

Figure 3a shows the GRs of all test meals. No significant difference at the baseline (i.e., 0 min) was found either in blood glucose levels or insulin among all the test meals. HR elicited significantly lower glucose levels at 30 and 45 min compared with R and SR, while it elicited reduced 30 min glucose compared with R. The eat-before-rice groups had consistent lower glucose concentrations during the first 45 min, and higher glucose concentrations at 120 min than their co-ingestion counterparts. The two eat-before-rice groups showed significant higher glucose level than R did at 180 min. However, there was no significant difference between HR and SR groups.

Figure 3b presents the insulin responses of all test meals. Compared with the H+R and S+R, the R, HR and SR had significantly higher insulin levels at 30 min. At 45 min, the SR showed significantly higher value of insulin than S+R and H+R did. There was a tendency for higher insulin concentrations between R and S+R (*p* = 0.087) and between the HR and H+R (*p* = 0.067). The H+R resulted a significant increase in insulin secretion compared with HR and R at 90 min, 120 min, 180 min. The S+R had higher insulin concentrations than R at 120 min and 180 min, while no significant difference was found between S+R and the co-ingestion meals at 90 min, 120 min, 180 min.

### 3.5. Postprandial Glucose and Insulin Response Characteristics

**Figure 4:**
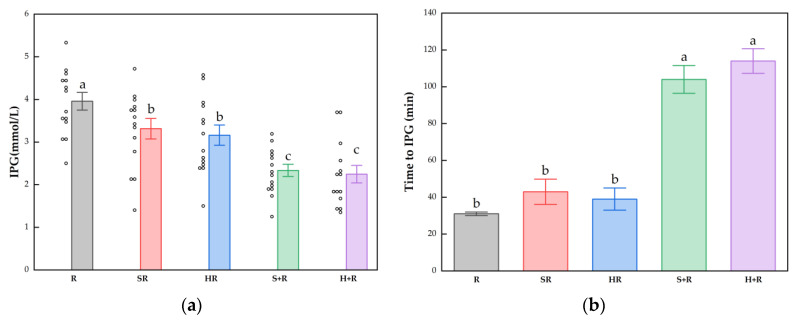

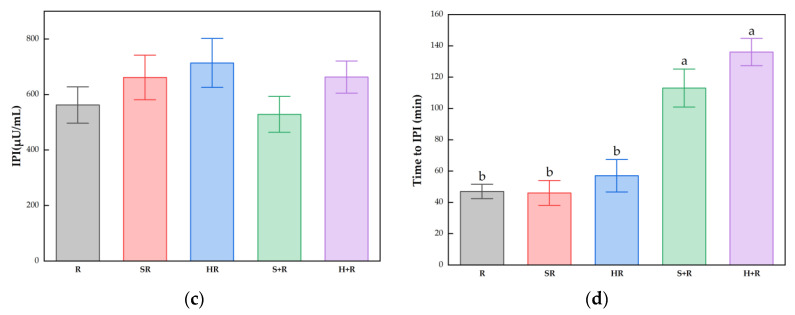
(**a**) IPG; (**b**) Time to IPG; (**c**) IPI; (**d**) Time to IPI. The hollow circles indicate the data of each subject, the columns indicate the mean value, the error bar indicates the SE value. Different letters indicate significant differences between means (*p* < 0.05). IPG, incremental peak glucose. IPI, incremental peak of insulin. R, rice; SR, co-ingestion of soft broccoli and rice; HR, co-ingestion of hard broccoli and rice; S+R, soft broccoli ingested prior to rice; H+R, hard broccoli ingested prior to rice. Different letters indicate significant differences between means (*p* < 0.05).

The IPG, time to IPG, IPI and time to IPI for all test meals are summarized in Figure 4. All the broccoli-containing meals decreased the glycemic peak significantly compared with R, while no significant difference was found between any test meals in terms of IPI, as shown in Figure 3. The SR and HR reduced the glycemic peaks by 15% and 20% compared with R (*p* < 0.05), respectively. The S+R and H+R further cut the glucose peak by 30% and 31%, compared with SR and HR (*p* < 0.05), respectively. Ingesting broccoli before rice delayed the times to reach glucose and insulin peak value, regardless of the texture of broccoli.

**Figure 5:**
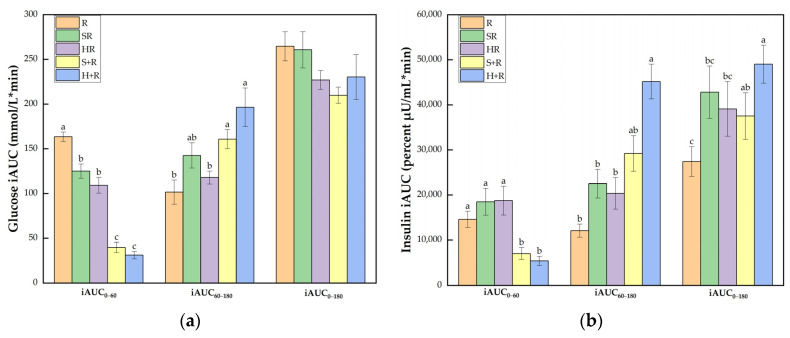
(**a**) Glucose iAUC; (**b**) Insulin iAUC. The columns indicate the mean value, the error bar indicates the SE value. Different letters indicate significant differences between means (*p* < 0.05). R, rice; SR, co-ingestion of soft broccoli and rice; HR, co-ingestion of hard broccoli and rice; S+R, soft broccoli ingested prior to rice; H+R, hard broccoli ingested prior to rice. iAUC, incremental areas under the curve. Different letters indicate significant differences between means (*p* < 0.05).

Figure 5a shows that all the broccoli-containing test meals achieved smaller glucose iAUC_0–60_ than R did (*p* < 0.001). The eat-before-rice groups elicited smaller iAUC_0–60_ than the co-ingestion group did, but had elevated glucose iAUC_60–180_ compared with HR and R (*p* < 0.05). The broccoli meals showed comparable iAUC_0–180_ compared with the rice reference while S+R showed a trend of decrease compared with R (*p* = 0.073).

As illustrated in Figure 5b, the eat-before-rice groups had significant smaller insulin iAUC_0–60_ than R while there was no difference between co-ingestion groups and R. In terms of iAUC_60–180_, H+R resulted in a significant larger area than R, SR and HR. The iAUC_0–180_ of H+R was significantly higher than any other test meals except for a trend of a 38% increase compared with S+R (*p* = 0.077). SR also tended to incur a 56% increase compared with R (*p* = 0.083).

### 3.6. Insulin Sensitivity

**Figure 6:**
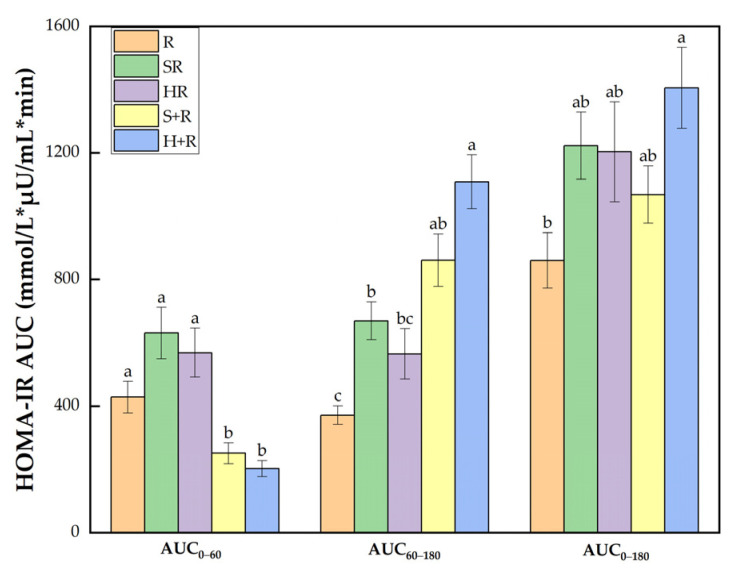
HOMA-IR AUC. The columns indicate the mean value, the error bar indicates the SE value. Different letters indicate significant differences between means (*p* < 0.05). R, rice; SR, co-ingestion of soft broccoli and rice; HR, co-ingestion of hard broccoli and rice; S+R, soft broccoli ingested prior to rice; H+R, hard broccoli ingested prior to rice. HOMA-IR AUC, homeostatic model areas under the curve. Different letters indicate significant differences between means (*p* < 0.05).

As shown in Figure 6, there was a significant group difference in HOMA-IR AUC_0–60_ between test meals. During 60–180 min, H+R had a HOMA-IR AUC 2.98 and was 1.65 times higher than R and HR, respectively (*p* < 0.05). SR and S+R had an 80% and 132% increase compared with R (*p* < 0.05). Throughout the session, the HOMA-IR AUC_0–180_ of H+R was 63% higher than that of R.

## 4. Discussion

In the current study, the addition of both soft and hard broccoli significantly curbed the postprandial glycemic excursion of a rice meal and impacted the postprandial insulin response in young health females in both the co-ingestion and preload pattern. The S+R halved IPG with comparable insulin sensitivity compared with the rice reference.

Broccoli was chosen in the present study for several reasons. It is a well-accepted nutrient-dense cruciferous vegetable associated with reduced risk of diabetes [5]. Broccoli is abundant in vitamin C, polyphenols such as flavanol glycosides, hydroxycinnamic acids, caffeic acid and ferulic acid [44] which are beneficial to the prevention and management of diabetes in the long run [45]. Broccoli is also a rich source of glucosinolates which improves obesity-induced insulin resistance by activating Nrf2 [46]. Daily intake of sulforaphane-concentrated broccoli sprout extract reduced fasting blood glucose and glycated hemoglobin (HbA1c) [47], decreased serum insulin concentration and HOMA-IR [48] in type 2 diabetic patients. Besides, it is not difficult to modify the texture of broccoli florets to achieve a proper chewiness by changing the cooking time.

Modifications of food texture have been shown to affect oral processing behaviors [28,49]. The hard broccoli contributed to a 26% increase of total mastication time, 17 additional chews and a significantly slower eating rate compared with the soft broccoli. This finding is in line with previous studies that demonstrated the association between food hardness and mastication behaviors [50,51].

Inconsistent to the Hypothesis 1, however, in the co-ingestion test group, no significant difference was observed in terms of the IPG, the time to IPG, the iAUC of glucose response, and the insulin responses except for the glucose value at 60 min between SR and HR. That might be partly explained by the fact that the acceptance of HR and SR was at the same level. The texture difference might not be as great as to induce significant physiological effect when co-ingested with rice. It is suggested that the texture variation of non-carbohydrate foods may not make a significant difference on postprandial GR and IR as the texture variation of carbohydrate foods did [52,53,54]. Furthermore, the effect of the mastication might be overshadowed by individual variation in the capacity of oral processing and the favorite hardness of cooked broccoli.

As expected, the combination of “eat-before-rice” pattern and texture-induced extra mastication achieved remarkably in terms of reducing the postprandial glycemic excursion. Compared with their co-ingested counterparts, the S+R and H+R further attenuated glucose response by reducing the IPG for 30% and 31%, and cut the iAUC_0–60_ for 68% and 72%, respectively. Again, there was no significant difference in terms of the hypoglycemic effect between the S+R and H+R.

In previous studies, the insulin responses with regard to vegetables and OSE were either improved or comparable to the reference, which is consistent with the result of the two co-ingestion meals and the S+R in our study. Adding green vegetables to potato or rice meals attenuated IR [55,56]. Broccoli consumed with mashed potato lowered the IPG with comparable IPI and though the broccoli-potato meal had decreased insulin iAUC_0–30_, a time-delayed lag in IR resulted in similar insulin iAUC_0–120_ compared with the potato meal [57]. In a fast-food style meal study, the addition of romaine lettuce reduced GR and IR while watercress induced similar IR compared with the control meal [58]. Boiled spinach added to a fat-rich bread and butter meal had no significant difference in IR compared with the bread meal [59].

However, in the present study, the H+R stimulated a 38% higher insulin iAUC (*p* = 0.077) than the S+R with a significant higher HOMA-IR AUC compared with the rice, though no difference was found in peak insulin values among any of the test meals and rice reference. This result suggests that the insulin response might be more susceptible to increased mastication induced by chewy texture when the vegetable was taken as a preload than as a co-ingestion food.

The mechanisms whereby vegetables could impact on acute postprandial GR might be explained as follows: (1) the polyphenols and phenolic acids [60] could inhibit digestive enzymes [61], such as pancreatic α-amylase [62], the Na^+^-glucose co-transporter [63], and thus modulate carbohydrate metabolism [64]; (2) the fibers could increase viscosity, delay gastric emptying, reduce the accessibility to enzymes, and inhibit amylase activity [65]; (3) the solid bulk of the hydrated vegetable might delay the gastric emptying as well as the absorption of glucose into the blood [57]. Nevertheless, none of the above mechanisms provides an explanation for the possible action of preload broccoli on insulin response in the current study.

In this study, 250 g vegetable was administered, much more than that used in the above-mentioned and the two rice-based trials containing green vegetables (120–164 g) [7,55,57]. When more vegetable was added to a fixed rice meal, especially as preload, the characteristics of the vegetable would play more important roles in IR, including the texture, the modified nutrient composition of the test meals and the presence of phytochemicals in the vegetable.

The preparation of vegetables might be a key factor for its physiological outcome. It was observed that the steaming time rather than the mastication behavior was associated with the oral hydrolysis and the physiological impact of glucosinolates [66]. The sulforaphanes in broccoli were reported to be able to reduce oxidative stress by the activation of antioxidant response pathways and consequently prevent insulin resistance [67]. It is mentioned that well steamed broccoli had the highest sulforaphane concentration with the highest hydrolysis rate of glucoraphanin. This is consistent with the current result that the soft broccoli performed better than the hard broccoli in the preload pattern. In the trial of the broccoli-potato meals [57], broccoli was steamed for 30 min and re-boiled for 11 min in a vacuum bag prior to consumption, and the extremely soft broccoli achieved a decreased IPG and insulin iAUC_0–30_. Addition of broccoli and spinach (boiled for 1 min in water) to a mixed meal (rice, tofu, egg and mayonnaise) lowered glucose concentration of 60 and 120 min with comparable IR compared with mixed meal consumption [36]. However, it is difficult to compare the OSE characteristics of the vegetables among the studies because the texture data and mastication behaviors were not available.

On the other hand, longer mastication could stimulate greater postprandial insulin response by up-regulating the activity of taste receptor, irrespective of the plasma glucose levels [68]. In this way, extra oral processing induced by large amount of broccoli might have a potential to reduce acute postprandial glucose peak by increasing insulin secretion. There is a possibility that, after consuming the hard broccoli prior to the rice meal, increased oral processing enhanced the release of glucagon-like peptide-1(GLP-1) [69], while the delayed digestion of carbohydrate resulted in slow-rising blood glucose concentration in the vegetable preload meal. The imbalance between the blood glucose and the incretin level might further lead to a relative postprandial hyperinsulinemia. Considering the healthy benefit of green vegetables such as broccoli, the slight acute increase of insulin concentration may not incur a major concern. However, in people of impaired insulin sensitivity, the long-term healthy outcomes of large amounts of chewy vegetables ingested before available carbohydrate foods need to be further tested.

Some studies reported that increased OSE might stimulate an early insulin response [31,70]. In the present study, no significant difference was observed, either between SR and HR, or between S+R and H+R, in terms of the time of insulin peak. This result did not support the Hypothesis 2, which expected that the HR with increased mastication would have earlier insulin secretion than SR did.

The insulin peaks seemed to synchronize with the glucose peaks in the test meals (30 min or 120 min). However, this result did not rule out the possibility of a difference of early cephalic phase insulin release (CPIR) as previous studies found [71,72], due to the 30 min interval of test time points in the present study. An early CPIR might not affect the time to insulin peak value, as the neurally-mediated, small and transient spike in insulin release is correlated with the magnitude of the postprandial insulin concentration [73]. What is more, in the preload meals, the slow digestion of carbohydrate induced by vegetable preload might delay the glucose and insulin peaks prior to the OSE effect [74,75].

To our knowledge, the current study is the first to explore the impact of oral processing induced by vegetables of different hardness on acute postprandial GR, IR and insulin sensitivity in both co-ingestion and preload pattern. In this study, the texture of cooked vegetable samples was determined by a texture analyzer, and the sensory acceptance was evaluated by a trained panel. The HR and SR were compared based on the same acceptability but different chewiness. A natural mastication paradigm was used to assess the effect of OSE variation in a real-life setting. The recruited participants in the trial were of the same ethnicity, gender, similar age and lifestyle to eliminate individual variability as much as possible. The result found that a combination of “eat-before-rice” pattern and texture-induced extra mastication might decrease insulin sensitivity instead of improving it.

Our study is an acute trial in healthy young female participants and the results may not apply to the obese, the prediabetic, the diabetic or male subjects. The early phase of insulin recruitment was not recorded, and the incretins such as GLP-1 were not determined. Considering the benefit of vegetables in chronic disease intervention and appetite control, the long-term modulating effect and the underlying mechanism of texture-induced oral processing of vegetables on insulin sensitivity need to be explored in future intervention studies.

## 5. Conclusions

The current study found that the addition of 250 g broccoli to a rice meal curbed the postprandial glycemic excursion in young health females, regardless of the co-ingested and preload mode. Taking broccoli prior to rice better reduced the IPG than the co-ingestion meals did. Taking the hard broccoli before rice resulted in a lowered postprandial insulin sensitivity compared with the rice reference, while the soft broccoli eaten-before-rice performed the best in halving the IPG without impairing the insulin sensitivity. Given that the “taking vegetables before high GI carbohydrate” strategy has been well accepted in postprandial glycemic management recently, the timing, the amount and the texture of vegetable food in a meal is worthy to be optimized in further studies.

## Figures and Tables

**Table 1:** Nutritional composition of test meals (per serving) ^1^.

Sample	Rice (g)	Broccoli (g)	AC ^2^ (g)	Protein (g)	Fat (g)	Dietary Fiber (g)	Weight ^3^ (g)	Energy (kcal)
R	171.5	-	50.0	7.0	0.6	1.00	478.9	845.6
SR	171.5	283.9	56.0	13.9	0.6	11.57	478.9	897.1
HR	171.5	307.4	56.0	13.9	0.6	11.57	478.9	897.1
S+R	171.5	283.9	56.0	13.9	0.6	11.57	478.9	897.1
H+R	171.5	307.4	56.0	13.9	0.6	11.57	478.9	897.1

^1^ Nutrient content data were acquired through manufacturers and determination experiments according to national standards; ^2^ AC, available carbohydrate; ^3^ The meal weight was balanced with additional plain water. The weight data of rice and broccoli are shown in a cooked condition. R, rice; SR, co-ingestion of soft broccoli and rice; HR, co-ingestion of hard broccoli and rice; S+R, soft broccoli ingested prior to rice; H+R, hard broccoli ingested prior to rice.

**Table 2:** Participant baseline characteristics.

Characteristics	Mean ± SD
Age (years)	22.8 ± 2.11
Weight (kg)	54.49 ± 7.05
Height (cm)	161.67 ± 4.22
BMI (kg/m^2^)	20.83 ± 2.31
Waist circumference (cm)	65.67 ± 3.75
Body fat (%)	27.93 ± 3.35
Fat-free mass (%)	67.88 ± 3.17
Systolic blood pressure (mmHg)	103.20 ± 8.65
Diastolic blood pressure (mmHg)	63.53 ± 7.58
Fasting blood glucose level (mmol/L)	4.73 ± 0.33
Fasting insulin level (µU/mL)	4.69 ± 0.92
Fasting HOMA-IR	0.99 ± 0.22

HOMA-IR, Homeostatic model.

**Table 3:** Instrumental texture characteristics of Broccoli.

Texture Characteristics	Raw Broccoli (R)	Soft Broccoli (SB)	Hard Broccoli (HB)
Puncture force (N)	8.48 ± 0.68 ^a^	0.79 ± 0.42 ^c^	4.47 ± 1.92 ^b^
Flexibility (mm)	1.50 ± 0.20 ^b^	2.06 ± 0.53 ^a^	1.84 ± 0.48 ^ab^
Shear force (N)	4.18 ± 0.93 ^b^	4.77 ± 0.86 ^b^	8.44 ± 1.70 ^a^
Toughness (N·mm)	14.06 ± 1.83 ^a^	13.18 ± 3.08 ^ab^	10.26 ± 3.02 ^b^
Brittleness	5.22 ± 0.97 ^a^	1.73 ± 0.59 ^c^	3.27 ± 0.79 ^b^

Different letters indicate significant differences between means (*p* < 0.05).

**Table 4:** Oral processing behaviors for rice and broccoli ^1^.

Oral Processing Behaviors	Rice (R)	Soft Broccoli (SB)	Hard Broccoli (HB)
Total mastication time (s)	23.36 ± 2.53 ^b^	29.45 ± 2.48 ^b^	37.11 ± 3.05 ^a^
Chew rate (chews/s)	1.54 ± 0.07	1.53 ± 0.04	1.59 ± 0.05
Number of chews (no.)	37.00 ± 4.69 ^b^	45.50 ± 4.43 ^b^	62.90 ± 6.16 ^a^
Chews per gram (no.) ^2^	7.44 ± 0.94 ^a^	4.37 ± 0.43 ^b^	5.89 ± 0.58 ^ab^
Eating rate (g/min)	14.37 ± 1.51 ^b^	22.35 ± 1.80 ^a^	17.51 ± 1.49 ^b^

^1^ Data are presented as Mean ± SEMs (*n* = 14 subjects, in duplicate); ^2^ Rice was served at a weight of 5.0 g and broccoli was served at a weight of 10.0 g. Different letters indicate significant differences between means (*p* < 0.05).

## Data Availability

The data presented in this study are available on request from the corresponding author.

## References

[1] Kolb H., Eizirik D.L. (2011). Resistance to type 2 diabetes mellitus: A matter of hormesis?. Nat. Rev. Endocrinol..

[2] Zheng J.S., Sharp S.J., Imamura F., Chowdhury R., Gundersen T.E., Steur M., Sluijs I., van der Schouw Y.T., Agudo A., Aune D. (2020). Association of plasma biomarkers of fruit and vegetable intake with incident type 2 diabetes: EPIC-InterAct case-cohort study in eight European countries. BMJ.

[3] Mamluk L., O’Doherty M.G., Orfanos P., Saitakis G., Woodside J.V., Liao L.M., Sinha R., Boffetta P., Trichopoulou A., Kee F. (2017). Fruit and vegetable intake and risk of incident of type 2 diabetes: Results from the consortium on health and ageing network of cohorts in Europe and the United States (CHANCES). Eur. J. Clin. Nutr..

[4] Wang P.Y., Fang J.C., Gao Z.H., Zhang C., Xie S.Y. (2016). Higher intake of fruits, vegetables or their fiber reduces the risk of type 2 diabetes: A meta-analysis. J. Diabetes Investig..

[5] Jia X., Zhong L., Song Y., Hu Y., Wang G., Sun S. (2016). Consumption of citrus and cruciferous vegetables with incident type 2 diabetes mellitus based on a meta-analysis of prospective study. Prim. Care Diabetes.

[6] Chiao-Hsin Y., Chia-Wei C., Jenshinn L. (2017). White Rice Glycemic Index Measured in Venous and Capillary Blood Samples. Food Sci. Technol. Res..

[7] Gustafsson K., Asp N.G., Hagander B., Nyman M. (1993). Effects of different vegetables in mixed meals on glucose homeostasis and satiety. Eur. J. Clin. Nutr..

[8] Imai S., Matsuda M., Hasegawa G., Fukui M., Obayashi H., Ozasa N., Kajiyama S. (2011). A simple meal plan of ‘eating vegetables before carbohydrate’ was more effective for achieving glycemic control than an exchange-based meal plan in Japanese patients with type 2 diabetes. Asia Pac. J. Clin. Nutr..

[9] Gopirajah R., Raichurkar K.P., Wadhwa R., Anandharamakrishnan C. (2016). The glycemic response to fibre rich foods and their relationship with gastric emptying and motor functions: An MRI study. Food Funct..

[10] Argyri K., Sotiropoulos A., Psarou E., Papazafiropoulou A., Zampelas A., Kapsokefalou M. (2013). Dessert formulation using sucralose and dextrin affects favorably postprandial response to glucose, insulin, and C-peptide in type 2 diabetic patients. Rev. Diabet. Stud..

[11] Coe S., Ryan L. (2016). Impact of polyphenol-rich sources on acute postprandial glycaemia: A systematic review. J. Nutr. Sci..

[12] Della G.L., Thomas M.A., Cena H. (2018). Insulin Sensitivity and Glucose Homeostasis Can Be Influenced by Metabolic Acid Load. Nutrients.

[13] Della G.L., Roggi C., Cena H. (2016). Diet-induced acidosis and alkali supplementation. Int. J. Food Sci. Nutr..

[14] Zhu R., Liu M., Han Y., Wang L., Ye T., Lu J., Fan Z. (2018). Acute effects of non-homogenised and homogenised vegetables added to rice-based meals on postprandial glycaemic responses and in vitro carbohydrate digestion. Br. J. Nutr..

[15] Engelen L., Fontijn-Tekamp A., van der Bilt A. (2005). The influence of product and oral characteristics on swallowing. Arch. Oral Biol..

[16] Ranawana V., Monro J.A., Mishra S., Henry C.J. (2010). Degree of particle size breakdown during mastication may be a possible cause of interindividual glycemic variability. Nutr. Res..

[17] Zhu Y., Hsu W.H., Hollis J.H. (2014). Increased number of chews during a fixed-amount meal suppresses postprandial appetite and modulates glycemic response in older males. Physiol. Behav..

[18] Madhu V., Shirali A., Pawaskar P.N., Madi D., Chowta N., Ramapuram J.T. (2016). Mastication Frequency and Postprandial Blood Sugar Levels in Normoglycaemic and Dysglycaemic Individuals: A Cross-Sectional Comparative Study. J. Clin. Diagn. Res..

[19] Zhu Y., Hsu W.H., Hollis J.H. (2013). Increasing the number of masticatory cycles is associated with reduced appetite and altered postprandial plasma concentrations of gut hormones, insulin and glucose. Br. J. Nutr..

[20] Slyper A. (2021). Oral Processing, Satiation and Obesity: Overview and Hypotheses. Diabetes Metab. Syndr. Obes..

[21] Read N.W., Welch I.M., Austen C.J., Barnish C., Bartlett C.E., Baxter A.J., Brown G., Compton M.E., Hume K.E., Storie I. (1986). Swallowing food without chewing; a simple way to reduce postprandial glycaemia. Br. J. Nutr..

[22] Ranawana V., Leow M.K., Henry C.J. (2014). Mastication effects on the glycaemic index: Impact on variability and practical implications. Eur. J. Clin. Nutr..

[23] Sato A., Ohtsuka Y., Yamanaka Y. (2019). Morning Mastication Enhances Postprandial Glucose Metabolism in Healthy Young Subjects. Tohoku J. Exp. Med..

[24] Tan V.M.H., Ooi D.S.Q., Kapur J., Wu T., Chan Y.H., Henry C.J., Lee Y.S. (2016). The role of digestive factors in determining glycemic response in a multiethnic Asian population. Eur. J. Nutr..

[25] Aguayo-Mendoza M.G., Ketel E.C., van der Linden E., Forde C.G., Piqueras-Fiszman B., Stieger M. (2019). Oral processing behavior of drinkable, spoonable and chewable foods is primarily determined by rheological and mechanical food properties. Food Qual. Prefer..

[26] Forde C.G., Leong C., Chia-Ming E., McCrickerd K. (2017). Fast or slow-foods? Describing natural variations in oral processing characteristics across a wide range of Asian foods. Food Funct..

[27] Mosca A.C., Torres A.P., Slob E., de Graaf K., McEwan J.A., Stieger M. (2019). Small food texture modifications can be used to change oral processing behaviour and to control ad libitum food intake. Appetite.

[28] McCrickerd K., Lim C.M.H., Leong C., Chia E.M., Forde C.G. (2017). Texture-Based Differences in Eating Rate Reduce the Impact of Increased Energy Density and Large Portions on Meal Size in Adults. J. Nutr..

[29] De Wijk R.A., Zijlstra N., Mars M., de Graaf C., Prinz J.F. (2008). The effects of food viscosity on bite size, bite effort and food intake. Physiol. Behav..

[30] Goh A.T., Choy J., Chua X.H., Ponnalagu S., Khoo C.M., Whitton C., van Dam R.M., Forde C.G. (2021). Increased oral processing and a slower eating rate increase glycaemic, insulin and satiety responses to a mixed meal tolerance test. Eur. J. Nutr..

[31] Laboure H., Van Wymelbeke V., Fantino M., Nicolaidis S. (2002). Behavioral, plasma, and calorimetric changes related to food texture modification in men. Am. J. Physiol. Regul. Integr. Comp. Physiol..

[32] Sun L., Goh H.J., Govindharajulu P., Leow M.K., Henry C.J. (2020). Postprandial glucose, insulin and incretin responses differ by test meal macronutrient ingestion sequence (PATTERN study). Clin. Nutr..

[33] Shukla A.P., Dickison M., Coughlin N., Karan A., Mauer E., Truong W., Casper A., Emiliano A.B., Kumar R.B., Saunders K.H. (2019). The impact of food order on postprandial glycaemic excursions in prediabetes. Diabetes Obes. Metab..

[34] Shukla A.P., Andono J., Touhamy S.H., Casper A., Iliescu R.G., Mauer E., Shan Z.Y., Ludwig D.S., Aronne L.J. (2017). Carbohydrate-last meal pattern lowers postprandial glucose and insulin excursions in type 2 diabetes. BMJ Open Diabetes Res. Care.

[35] Bhavadharini B., Mohan V., Dehghan M., Rangarajan S., Swaminathan S., Rosengren A., Wielgosz A., Avezum A., Lopez-Jaramillo P., Lanas F. (2020). White Rice Intake and Incident Diabetes: A Study of 132,373 Participants in 21 Countries. Diabetes Care.

[36] Kameyama N., Maruyama C., Matsui S., Araki R., Yamada Y., Maruyama T. (2014). Effects of consumption of main and side dishes with white rice on postprandial glucose, insulin, glucose-dependent insulinotropic polypeptide and glucagon-like peptide-1 responses in healthy Japanese men. Br. J. Nutr..

[37] Ranawana V., Clegg M.E., Shafat A., Henry C.J. (2011). Postmastication digestion factors influence glycemic variability in humans. Nutr. Res..

[38] Barrett D.M., Garcia E., Wayne J.E. (1998). Textural modification of processing tomatoes. Crit. Rev. Food Sci. Nutr..

[39] Van Eck A., Wijne C., Fogliano V., Stieger M., Scholten E. (2019). Shape up! How shape, size and addition of condiments influence eating behavior towards vegetables. Food Funct..

[40] Wolever T.M. (2004). Effect of blood sampling schedule and method of calculating the area under the curve on validity and precision of glycaemic index values. Br. J. Nutr..

[41] Matthews D.R., Hosker J.P., Rudenski A.S., Naylor B.A., Treacher D.F., Turner R.C. (1985). Homeostasis model assessment: Insulin resistance and beta-cell function from fasting plasma glucose and insulin concentrations in man. Diabetologia.

[42] Borer K.T., Lin P.J., Wuorinen E. (2021). Timing of Meals and Exercise Affects Hormonal Control of Glucoregulation, Insulin Resistance, Substrate Metabolism, and Gastrointestinal Hormones, but Has Little Effect on Appetite in Postmenopausal Women. Nutrients.

[43] Wolever T.M.S., Brand-Miller J.C., Abernethy J., Astrup A., Atkinson F., Axelsen M., Bjorck I., Brighenti F., Brown R., Brynes A. (2008). Measuring the glycemic index of foods: Interlaboratory study. Am. J. Clin. Nutr..

[44] Vallejo F., Tomas-Barberan F.A., Ferreres F. (2004). Characterisation of flavonols in broccoli (Brassica oleracea L. var. italica) by liquid chromatography-uV diode-array detection-electrospray ionisation mass spectrometry. J. Chromatogr. A.

[45] Cao H., Ou J., Chen L., Zhang Y., Szkudelski T., Delmas D., Daglia M., Xiao J. (2019). Dietary polyphenols and type 2 diabetes: Human Study and Clinical Trial. Crit. Rev. Food Sci. Nutr..

[46] Xu L., Nagata N., Ota T. (2018). Glucoraphanin: A broccoli sprout extract that ameliorates obesity-induced inflammation and insulin resistance. Adipocyte.

[47] Axelsson A.S., Tubbs E., Mecham B., Chacko S., Nenonen H.A., Tang Y., Fahey J.W., Derry J., Wollheim C.B., Wierup N. (2017). Sulforaphane reduces hepatic glucose production and improves glucose control in patients with type 2 diabetes. Sci. Transl. Med..

[48] Bahadoran Z., Tohidi M., Nazeri P., Mehran M., Azizi F., Mirmiran P. (2012). Effect of broccoli sprouts on insulin resistance in type 2 diabetic patients: A randomized double-blind clinical trial. Int. J. Food Sci. Nutr..

[49] Tonni I., Riccardi G., Piancino M.G., Stretti C., Costantinides F., Paganelli C. (2020). The influence of food hardness on the physiological parameters of mastication: A systematic review. Arch. Oral Biol..

[50] Bolhuis D.P., Forde C.G., Cheng Y., Xu H., Martin N., de Graaf C. (2014). Slow food: Sustained impact of harder foods on the reduction in energy intake over the course of the day. PLoS ONE.

[51] Zijlstra N., Mars M., Stafleu A., de Graaf C. (2010). The effect of texture differences on satiation in 3 pairs of solid foods. Appetite.

[52] Choy J., Goh A.T., Chatonidi G., Ponnalagu S., Wee S., Stieger M., Forde C.G. (2021). Impact of food texture modifications on oral processing behaviour, bolus properties and postprandial glucose responses. Curr. Res. Food Sci..

[53] Goh A.T., Chatonidi G., Choy M., Ponnalagu S., Stieger M., Forde C.G. (2021). Impact of Individual Differences in Eating Rate on Oral Processing, Bolus Properties and Post-Meal Glucose Responses. Physiol. Behav..

[54] McArthur B.M., Mattes R.D., Considine R.V. (2018). Mastication of Nuts under Realistic Eating Conditions: Implications for Energy Balance. Nutrients.

[55] Sun L., Ranawana D.V., Leow M.K., Henry C.J. (2014). Effect of chicken, fat and vegetable on glycaemia and insulinaemia to a white rice-based meal in healthy adults. Eur. J. Nutr..

[56] Hatonen K.A., Virtamo J., Eriksson J.G., Sinkko H.K., Sundvall J.E., Valsta L.M. (2011). Protein and fat modify the glycaemic and insulinaemic responses to a mashed potato-based meal. Br. J. Nutr..

[57] Ballance S., Knutsen S.H., Fosvold O.W., Wickham M., Trenado C.D., Monro J. (2018). Glyceamic and insulinaemic response to mashed potato alone, or with broccoli, broccoli fibre or cellulose in healthy adults. Eur. J. Nutr..

[58] Shokraei S., Khandouzi N., Sina Z., Nasrollahzadeh J. (2021). The acute effect of incorporating lettuce or watercress into a moderately high-fat meal on postprandial lipid, glycemic response, and plasma inflammatory cytokines in healthy young men: A randomized crossover trial. Lipids Health Dis..

[59] Maruyama C., Kikuchi N., Masuya Y., Hirota S., Araki R., Maruyama T. (2013). Effects of green-leafy vegetable intake on postprandial glycemic and lipidemic responses and alpha-tocopherol concentration in normal weight and obese men. J. Nutr. Sci. Vitaminol..

[60] Vallejo F., Tomas-Barberan F., Garcia-Viguera C. (2003). Health-promoting compounds in broccoli as influenced by refrigerated transport and retail sale period. J. Agr. Food Chem..

[61] Miao J., Li X., Zhao C., Gao X., Wang Y., Gao W. (2018). Active compounds, antioxidant activity and alpha-glucosidase inhibitory activity of different varieties of Chaenomeles fruits. Food Chem..

[62] Ogawa N., Satsu H., Watanabe H., Fukaya M., Tsukamoto Y., Miyamoto Y., Shimizu M. (2000). Acetic acid suppresses the increase in disaccharidase activity that occurs during culture of caco-2 cells. J. Nutr..

[63] Li J.M., Che C.T., Lau C.B., Leung P.S., Cheng C.H. (2006). Inhibition of intestinal and renal Na+-glucose cotransporter by naringenin. Int. J. Biochem. Cell Biol..

[64] Bahadoran Z., Mirmiran P., Azizi F. (2013). Dietary polyphenols as potential nutraceuticals in management of diabetes: A review. J. Diabetes Metab. Disord..

[65] Yu K., Ke M.Y., Li W.H., Zhang S.Q., Fang X.C. (2014). The impact of soluble dietary fibre on gastric emptying, postprandial blood glucose and insulin in patients with type 2 diabetes. Asia Pac. J. Clin. Nutr..

[66] Irmela S., Michelle V.D.K., Teresa O., Matthijs D., Ruud V. (2018). The effect of chewing on oral glucoraphanin hydrolysis in raw and steamed broccoli. J. Funct. Foods.

[67] Evans J.L. (2007). Antioxidants: Do they have a role in the treatment of insulin resistance?. Indian J. Med. Res..

[68] Katsarou V., Tsolaki M., Galanakis C.M. (2019). Trends in Personalized Nutrition. Personalized Nutrition by Predicting Glycemic Responses.

[69] Xu J., Xiao X., Li Y., Zheng J., Li W., Zhang Q., Wang Z. (2015). The effect of gum chewing on blood GLP-1 concentration in fasted, healthy, non-obese men. Endocrine.

[70] Lasschuijt M., Mars M., de Graaf C., Smeets P. (2020). How oro-sensory exposure and eating rate affect satiation and associated endocrine responses—A randomized trial. Am. J. Clin. Nutr..

[71] Just T., Pau H.W., Engel U., Hummel T. (2008). Cephalic phase insulin release in healthy humans after taste stimulation?. Appetite.

[72] Smeets P.A., Erkner A., de Graaf C. (2010). Cephalic phase responses and appetite. Nutr. Rev..

[73] Teff K.L., Mattes R.D., Engelman K., Mattern J. (1993). Cephalic-phase insulin in obese and normal-weight men: Relation to postprandial insulin. Metabolism.

[74] Imai S., Fukui M., Ozasa N., Ozeki T., Kurokawa M., Komatsu T., Kajiyama S. (2013). Eating vegetables before carbohydrates improves postprandial glucose excursions. Diabet. Med..

[75] Imai S., Fukui M., Kajiyama S. (2014). Effect of eating vegetables before carbohydrates on glucose excursions in patients with type 2 diabetes. J. Clin. Biochem. Nutr..

